# Gut microbiome composition and intestinal immunity in antiphospholipid syndrome patients versus healthy controls

**DOI:** 10.1177/09612033241274515

**Published:** 2024-08-17

**Authors:** Valérie LBI Jansen, Mark Davids, Dagmar JM van Mourik, Johannes HM Levels, Michiel Coppens, Saskia Middeldorp, Max Nieuwdorp, Thijs E van Mens

**Affiliations:** 1 Department of (Experimental) Vascular Medicine, 26066 Amsterdam UMC Location University of Amsterdam, Amsterdam, The Netherlands; 2 Amsterdam Cardiovascular Sciences, Pulmonary Hypertension & Thrombosis, Amsterdam, The Netherlands; 3 Amsterdam Reproduction & Development Research Institute, Amsterdam, The Netherlands; 4 Department of Medicine - Thrombosis and Haemostasis, 4501 Leiden University Medical Center, Leiden, The Netherlands; 5 Department of Internal Medicine, 6034 Radboud University Medical Center, Nijmegen, The Netherlands

**Keywords:** Antiphospholipid syndrome, lupus anticoagulant, Autoimmune disease, Gut microbiome, Intestinal microbiome

## Abstract

**Introduction:**

The gut microbiome is recognized as a factor that could potentially contribute to the persistent antibodies of antiphospholipid syndrome (APS). Gut microbial interventions can both induce and mitigate APS in mice. In human APS patients, anti-beta-2-glycoprotein I (β2GP-1) titers correlate with antibody titers against a gut commensal protein homologous to β2GP-1.

**Aim:**

To  investigate the effect of the intestinal microenvironment on human APS. Methods We cross-sectionally compared intestinal microbiota composition quantified by shotgun sequencing; fecal short chain fatty acids (SCFAs), bacterial metabolites known to affect autoimmune processes; and fecal calprotectin, an intestinal inflammatory marker, in APS patients and healthy controls.

**Results:**

Neither alpha nor beta diversity of the gut microbiota differed between APS patients (n = 15) and controls (n = 16) and no taxa were differentially abundant. Moreover, fecal SCFAs and fecal calprotectin, did not differ between the groups.

**Conclusion:**

Gut microbiome effects on the APS phenotype are likely not driven by bacterial overabundance, SCFA production or intestinal inflammation.

## Introduction

Antiphospholipid syndrome (APS) is an autoimmune disease that is characterized by thrombotic events or pregnancy morbidity in the presence of persistent antiphospholipid antibodies (APLAs).^1^ As for other autoimmune disorders, the origin of these antibodies is unknown. Transient antiphospholipid antibodies are a relatively prevalent phenomenon and have been associated with acute infectious triggers, such as HIV, HCV and EBV infections.^2^

The gut microbiota, the collection of microorganisms residing in the gut, pose a chronic exposure to an extensive diversity of antigens and are hypothesized to contribute to the formation of persistent antibodies and development of APS.^3^ An unbalanced composition of the gut microbiota and ensuing low grade inflammation of the intestinal wall could potentially lead to loss of peripheral tolerance by inducing the required pro-inflammatory microenvironment to co-stimulate lymphocytes.^3^ Moreover, molecular mimicry, which is the homology between microbial peptides and self-peptides, could lead to activation of cross-reactive lymphocytes and thus drive autoimmunity.^4^ Recently, in both APS and systemic lupus erythematosus (SLE) gut commensals have been identified that contain peptide sequences homologous to the associated autoantigens, β2GP-1 and Ro60, respectively.^5,6^

Peptide sequences homologous to the β2GP-1 epitopes were found in gut bacterium *Roseburia intestinalis (R. intestinalis)*.^5^ The APLA epitope homologue was identified in protein DNA methyl transferase. A monoclonal anti-β2GP-1 antibody as well as patient plasma-derived polyclonal IgG were shown to cross-react with *R. intestinalis* DNA methyl transferase.^5^ In vivo, both anti-β2GP-1 titers and the ischemic phenotype of (NZW × BXSB)F1 mice were dependent on the presence of *R. intestinalis* as oral administration of the bacterium induced β2GP-1 cross-reactive antibodies and thrombotic events.^5^ Conversely, oral vancomycin treatment diminished anti-β2GP1 titers and thrombotic events and prolonged survival.^7^ As stated, the gut microbiota may in parallel interfere with intestinal inflammation, which could potentially contribute to autoimmunity. In this regard, calprotectin is derived from activated neutrophils and monocytes, and fecal calprotectin is a recognized biomarker for intestinal inflammation. Both in patients with SLE and APS, increased levels of fecal calprotectin have been demonstrated.^5,7^

In addition to observations supporting cross-reactivity, the gut microbiota are known to produce metabolites that signal to the immune system. Short chain fatty acids (SCFAs) are derived from the fermentation of dietary fiber by intestinal bacteria and can influence regulatory T cells as well as intestinal barrier function.^8^ In lupus-prone MRL/*Fas*^*lpr/lpr*^ mice, administration of SCFAs butyrate and propionate decreased autoantibody production and mitigated the SLE pathology.^9^ Supplementation of butyrate also diminished disease activity in a rheumatic arthritis mouse model.^10^ Furthermore, patients with rheumatoid arthritis show depleted levels of fecal propionate and butyrate compared to age- and sex-matched controls.^10^ However, fecal SCFAs have not been evaluated in patients with APS.

Murine studies and in vitro data indicate a role of gut microbiota composition, gut microbiota derived metabolites and intestinal inflammation in APS but high taxonomic resolution gut microbiota composition data and data on fecal SCFAs in APS patients are lacking.

The aim of the present study is to evaluate the effects of the intestinal microenvironment on human APS, by comparing gut microbiota composition and abundance of potentially cross-reactive microbes, intestinal inflammation and SCFA production in patients with APS and healthy controls.

## Methods

We included patients with APS who met the Sydney classification criteria^1^ and who tested positive for anti-β2GP-1 antibodies on their last APLA evaluation, considering the B and T cell epitope homologues to anti-β2GP-1 epitopes in *R. intestinalis.* Patients were recruited from a tertiary care vascular medicine department and through the Dutch patients association website. All patients had stable disease, defined by the absence of classifying events, both thrombotic and obstetric, for at least 3 months preceding inclusion. Controls were recruited from the patient’s social environment to ensure comparability at the level of the source population, but excluding relatives or household members. We excluded subjects with recent use of antibiotics or proton pump inhibitors because of their gut microbiota distorting effects. Given the vast overrepresentation of women in the eligible subjects, men were excluded to avoid that potential associations between exposure variables and APS were confounded by gender.

Written informed consent was obtained from all participants. The study was approved by the Amsterdam UMC Biobank Committee and conducted in accordance with the Declaration of Helsinki. Subjects collected a single fecal sample at home, no more than 24 h preceding the study visit. Samples were kept at 4°C until the study visit and were then stored at −80°C until further processing.

### Metagenomics

DNA was isolated from fecal samples using a bead beading method as previously decribed^11^ and microbiota composition was analyzed with shotgun metagenomics sequencing obtained by Illumina Novaseq 6000 sequencing (Novogene, Cambridge, UK).

Taxonomic profiles were determined using metaphlan3.^12^ Abundance of *R*. *intestinalis* DNA methyltranseferase was determined by aligning reads using bowtie2^13^ against the gene including a 200 bp upstream and downstream region.

We measured fecal SCFAs propionate, acetate, formate and butyrate using high-performance liquid chromatography with ultraviolet detection as previously described.^14^ For each sample SCFA measurements were corrected for differences in wet and dry weight. Calprotectin was measured by automated immunoassay using the EliA^TM^ Calprotectin 2 assay (Thermo Fisher Scientific, Utrecht, the Netherlands) on the Phadia 250 instrument. For SCFA and calprotectin measurements all samples were analyzed on the same day and in random order to avoid batch effects.

### Statistical analysis

Baseline characteristics were summarized with descriptive statistics. Categorical variables were compared with Fisher’s exact tests, numerical variables were compared using Mann-Whitney U tests. Fecal SCFA levels and fecal calprotectin levels were compared using Mann-Whitney U tests. SCFA level analyses were corrected for multiple testing with the Bonferroni method. Statistical analysis was performed in R (3.5). Alpha diversity metrics were calculated using phyloseq.^15^ Compositional differences were assessed using Bray-Curtis dissimilarity distance and tested using vegans adonis permanova function. Differential taxa abundance was tested using ANCOMBC.^16^

## Results

We included 15 patients and 16 controls [Table 1]. The cohort comprised both obstetric and thrombotic APS patients, with a predominance of obstetric manifestations. There were no significant differences in age and ethnicity between the two groups. 53% of APS patients used anticoagulant medication and 20% used antiplatelet therapy. The controls did not.

**Table 1.** Characteristics of included APS patients and healthy controls.

	APS patients *n* = 15	Healthy controls *n* = 16	*p*-value
Age, median (IQR)	34.0 (31.0, 43.5)	39.5 (32.8, 49.0)	.464
BMI, median (IQR)	23.8 (21.9, 26.3)	23.3 (21.5, 27.1)	.934
Ethnicity, *n* (%)			.226
Caucasian	13 (86.7)	16 (100.0)	
Mixed	1 (6.7)	0 (0.0)	
Other	1 (6.7)	0 (0.0)	
Obstetric history, *n* (%)			
Gravidity, mean ± SD	3.5 ± 1.3	2.5 ± 1.0	.060
Fetal loss >10 weeks of gestation	9 (60.0)	1 (6.2)	.001
≥3 early pregnancy losses	1 (6.7)	0 (0.0)	.528
Pre-term birth <34 weeks of gestation	1 (6.7)	0 (0.0)	.528
History of thrombosis, *n* (%)			
Venous thrombosis	6 (40.0)	1 (6.2)	.037
Arterial thrombosis	4 (26.7)	0 (0.0)	.043
APLA positivity at diagnosis^a^, *n* (%)			
Lupus anticoagulant	6 (40.0)	NA	
Anti-beta-2-glycoprotein-I IgG	7 (46.7)	NA	
Anti-beta-2-glycoprotein-I IgM	2 (13.3)	NA	
Anti-cardiolipin IgG	7 (46.7)	NA	
Anti-cardiolipin IgM	1 (6.7)	NA	
Triple positive	2 (13.3)	NA	
SLE, *n* (%)	2 (13.3)	0 (0.0)	.101
Medication, *n* (%)			
Glucocorticoid	0 (0.0)	0 (0.0)	NA
Antimalarial	1 (6.7)	0 (0.0)	.484
Antiplatelet therapy	3 (20.0)	0 (0.0)	.101
Anticoagulants, *n* (%)			
VKA	4 (26.7)	0 (0.0)	.043
DOAC	4 (26.7)	0 (0.0)	.043
None	7 (46.6)	16 (100)	.001

APS: antiphospholipid syndrome, defined by Sydney criteria; IQR: interquartile range; BMI: body mass index; SD: standard deviation; APLA: antiphospholipid antibody; SLE systemic lupus erythematosus; VKA: vitamin K antagonist; DOAC: direct oral anticoagulants.

^a^defined as meeting the Sydney criteria for the particular APLA.

### Gut microbiota composition

**Figure 1.**
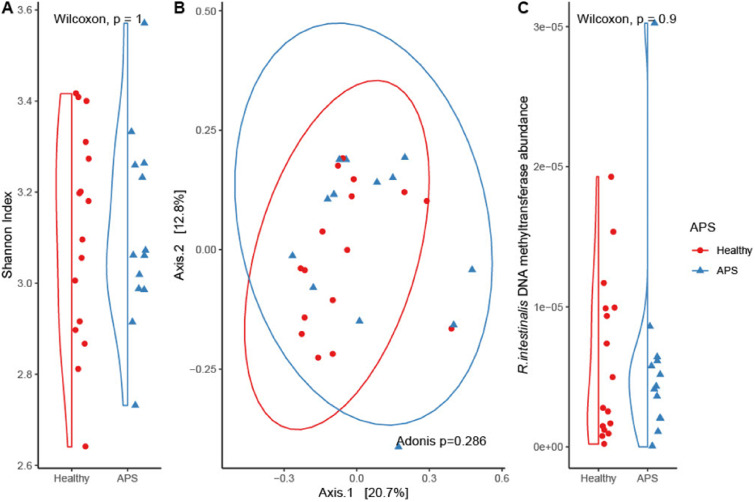
Gut microbiota in APS patients and controls (A) alpha diversity assessed by Shannon index (B) beta diversity: PCoA of between sample Bray-Curtis dissimilarity (C) *R. intestinalis* DNA methyltransferase gene abundance. APS: antiphospholipid syndrome; PCoA: principal coordinates analysis; *R. intestinalis: Roseburia intestinalis*.

We found no differences in alpha diversity, quantified by Shannon Index, between APS patients and healthy controls (Figure 1A). Principal coordinate Analysis (PCoA) of beta diversity, assessed by Bray-Curtis dissimilarity, did not differentiate APS patients from healthy controls (Figure 1B). *R. intestinalis* DNA methyl transferase was not more abundant in APS patients (Figure 1C). Differential abundance testing using ANCOMBC did not reveal any taxa associations.

### Fecal short chain fatty acid and calprotectin levels

**Figure 2.**
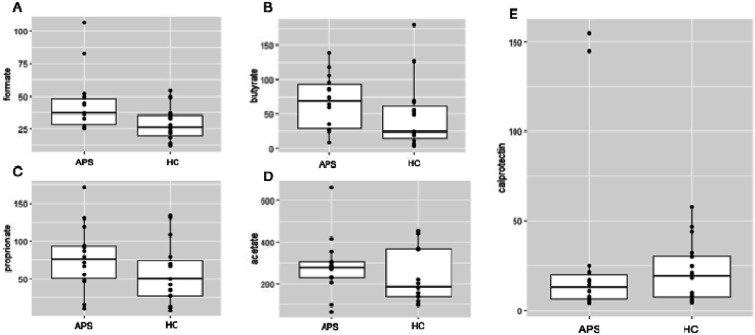
Fecal short chain fatty acids concentrations in µmol/g corrected for differences in wet and dry weight (A) formate (B) butyrate (C) propionate (D) acetate (E) calprotectin levels in μg/g. APS: antiphospholipid syndrome; HC: healthy controls.

There were no differences in fecal SCFAs levels between patients and controls. One control was excluded from SCFAs level measurement because the amount of sample was insufficient. APS patients had numerically higher fecal formate concentrations compared to controls, median 36.9 μmol/g (IQR 28.3–48.0) versus 25.7 μmol/g (IQR 20.1–35.4), but this was not significant after correcting for multiple testing (adjusted *p*-value .064) (Figure 2A). We found no differences in acetate, butyrate or propionate concentration (Figure 2B–D).

Fecal calprotectin levels did not differ between APS patients and controls, median 12.5 mg/kg (IQR 6.3–20.0) and 19.5 mg/kg (IQR 7.5–30.5), respectively (*p* = .492).

## Discussion

In the current study, we attempted to corroborate preclinical findings suggesting a possible role of the intestinal microenvironment in human APS. Our high taxonomic resolution data showed no difference in gut microbiota composition in a cohort of APS patients compared to controls.

Previous data on gut microbiota composition are conflicting, with one study showing that the *Collinsella* genus and the *Bifidobacterium*^5^ genus were more abundant in APS patients compared to controls whereas another found no differences in gut microbiota composition between APS patients and controls.^5,17^ Despite its apparent central role in the pathophysiology of experimental murine APS, *R. intestinalis* DNA methyl transferase was not more abundant in our study, which is in line with previous 16S data.^5^

We found no differences in fecal SCFAs between APS patients and healthy controls in spite of data on the role of SCFA in murine SLE.^9^ To what extent fecal SCFA levels reflect SCFA production in the gut remains somewhat uncertain however, considering SCFAs are predominantly absorbed and further metabolised.^18^ We also found no evidence of intestinal inflammation in our cohort, contrary to findings of increased fecal calprotectin levels in APS patients in a previous study.^5^

Ultimately, preclinical data on the effect of gut microbiota on APS and related autoimmune diseases did not translate into differences in gut microbiota composition or intestinal inflammation in human APS. The influence of gut microbes may be mediated by specific immunological response to these microbes in concert with other environmental and host factors such as HLA-allotype, rather than simple abundance or general intestinal inflammation. This is underlined by data on intestinal IgA responses to gut microbes in patients with APS that showed that within the IgA coated fraction of the gut microbiome, both alpha and beta diversity differed between APS patients and healthy controls.^5^

Strengths of our study include a well-defined cohort of APS patients, diagnosed according to Sydney research criteria.^1^ Controls were selected from the social environment of patients to limit differences in confounding variables such as geography, lifestyle, diet and socio-economic status. Moreover, gut microbiota composition was assessed using shotgun metagenomic sequencing, which provides higher genomic resolution compared to 16s sequencing used in earlier studies.

Our study has certain limitations that also warrant comment. Given the modest sample size lack of statistical power and type II errors cannot be excluded. In the analyses of SCFA levels Bonferroni correction for multiple testing was needed to avoid false positives but this method also reduces statistical power.

The cross-sectional design assumes stability of microbiota composition and other measurements over time. Indeed, the gut microbiota has been shown to be relatively stable throughout adulthood.^19^ Diet is a potential confounder in gut microbiota studies and dietary influences could not be evaluated because of limited data on dietary intake. Finally, sex differences could not be investigated as men were excluded because too few men were eligible for the study. Consequently, the results of this study may not be extrapolated to men.

This study found no differences in gut microbiota composition, fecal SCFAs or fecal calprotectin in APS patients versus controls. Future studies should focus on gut microbiome modulation with antibiotics, probiotics or fecal microbiome transplantation. The therapeutic potential of gut microbiome directed interventions is underlined by a single arm study in 20 SLE patients showing that administration of healthy gut microbiome diminished disease activity and anti-dsDNA titers.^20^ Moreover, in order to develop specific gut microbiome directed therapies, more insight in the mechanisms underlying the role of the gut microbiome in APS is needed.

In conclusion, presence of specific microbes may be a prerequisite to develop persistent antiphospholipid syndrome but this development seems to be driven by other factors than microbiota abundance, SCFAs or general intestinal inflammation.

## Data Availability

Gut micrbiome sequencing, fecal short chain fatty acids and fecal calprotectin data are publicly accessible via: Mendeley Data, V1, doi: 10.17632/nssynknfxx.1.
